# First person – Mireia Sueca-Comes

**DOI:** 10.1242/dmm.052216

**Published:** 2024-12-13

## Abstract

First Person is a series of interviews with the first authors of a selection of papers published in Disease Models & Mechanisms, helping researchers promote themselves alongside their papers. Mireia Sueca-Comes is first author on ‘ The role of mesenchymal cells in cholangiocarcinoma’, published in DMM. Mireia conducted the research described in this article while a PhD student in Anna M. Grabowska's lab at University of Nottingham, Nottingham, UK. She is now a Client Account Manager at HistologiX, Nottingham, UK, where she designs study proposals for companies and universities around the world.


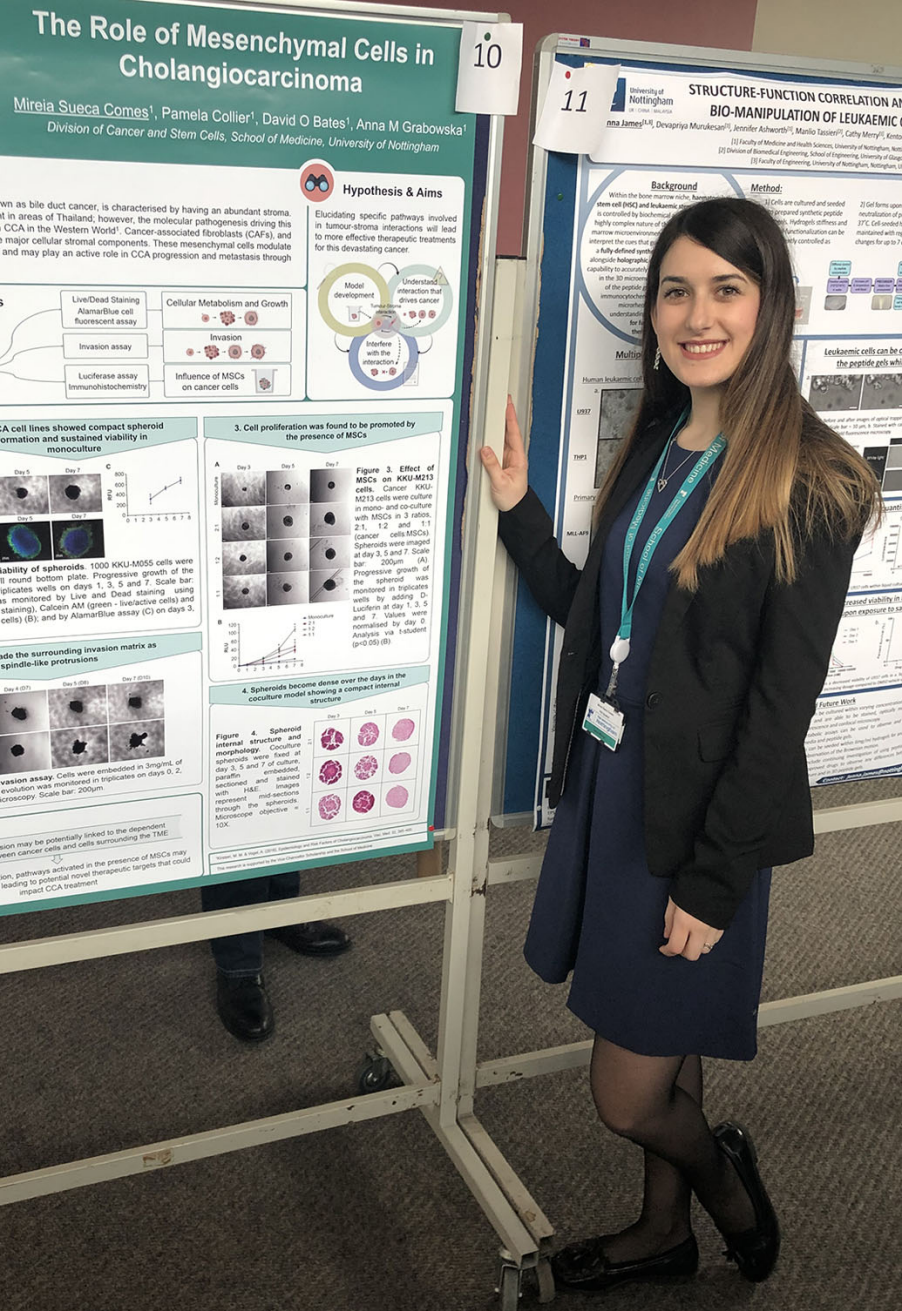


**Mireia Sueca-Comes**

**Who or what inspired you to become a scientist?**

My journey into science was shaped by personal experiences with cancer in my family, which sparked a deep curiosity from an early age. I was driven by a desire to understand how such a complex disease develops and affects the body, and this sense of curiosity set me on the path to explore biology and medical science. Over time, what started as a personal connection to the subject evolved into a fascination with the intricate ways the body responds to challenges like cancer.

As I began to learn more about the scientific community, I found inspiration in the dedication of researchers and the tangible impact of their work. Attending conferences and seeing how research could directly improve people's lives was particularly inspiring and reinforced my determination to be part of this field. It made me realise how many people depend on these discoveries and how important it is to continue pushing the boundaries of what we know.

I've always been captivated by the balance between health and disease, especially in the context of cancer and its interaction with the tumour microenvironment. The complexity of these systems drives my passion for research and motivates me to contribute to improving our understanding, with the hope of making a meaningful difference through science and ultimately helping patients and families who have unfortunately been through a similar situation to mine.

**What is the main question or challenge in disease biology you are addressing in this paper? How did you go about investigating your question or challenge?**

The main question addressed in this paper is how the tumour microenvironment (TME) influences the progression of cholangiocarcinoma (CCA), a desmoplastic and highly aggressive cancer with limited early biomarkers and effective treatments. While the importance of the TME in cancer progression is widely acknowledged, its specific contributions to CCA remain unclear, particularly in terms of the molecular mechanisms involved. This study aims to understand how the interaction between CCA cells and stromal cells, specifically mesenchymal stem cells (MSCs), impacts tumour growth and survival.

To explore this, I used patient-derived xenograft (PDX) models of CCA and corresponding patient samples, performing RNA sequencing to identify differentially expressed genes and pathways associated with disease progression. This approach allowed me to identify key dysregulated pathways that were activated in the presence of MSCs. To further investigate these findings, I developed spheroid models of CCA, both with and without MSCs, to more accurately mimic the TME. These models were analysed for changes in tumour morphology, growth and viability.

The results showed that the addition of MSCs restored critical signalling pathways, including cancer-associated kinase activity. This restoration led to enhanced tumour growth and survival, demonstrating the vital role of stromal components in tumour progression. By including MSCs in these models, this research highlights the importance of accurately modelling the TME to better understand its role in CCA and improve preclinical testing for potential therapies.

**How would you explain the main findings of your paper to non-scientific family and friends?**

My research looks at how the environment around a tumour affects the growth and spread of a specific type of cancer. One of the key players in this environment are cells called MSCs, which are part of the tumour's surrounding tissue.

What we found is that when we added these MSCs to cancer cell models, the tumour grew more quickly and became more resilient. This is because the MSCs help the cancer cells communicate better with their surroundings, essentially helping the tumour survive and grow. By studying this interaction, we've identified some important pathways in the cancer cells that could be targeted in future treatments.

This research is important because it shows how crucial the tumour's surroundings are in its development. By understanding these interactions more deeply, we can start to think about better ways to treat this kind of cancer by focusing not just on the tumour cells themselves, but on the environment around them as well.

[…] this research shows that the environment surrounding the tumour plays an active role in promoting tumour growth and survival.

**What are the potential implications of these results for disease biology and the possible impact on patients?**

The potential implications of these results for disease biology are significant, as they provide new insights into the role of the TME in the progression of CCA. By highlighting the crucial interactions between CCA cells and MSCs within the TME, this research shows that the environment surrounding the tumour plays an active role in promoting tumour growth and survival. This shifts the focus of cancer research from solely targeting the tumour cells themselves to also considering how the tumour's surrounding tissue influences its behaviour. Understanding these interactions could help identify new molecular pathways that drive tumour progression, which could ultimately lead to the development of more effective treatment strategies. Additionally, improving pre-clinical models of CCA to better replicate the TME could lead to more accurate testing of potential drugs and therapies, potentially speeding up the development of new treatments for this difficult-to-treat cancer.

For patients, these findings could have a significant impact on future therapies. By targeting the signalling pathways activated by MSCs in the TME, we may be able to disrupt the processes that help the tumour grow and become resistant to treatment.

**Figure:**
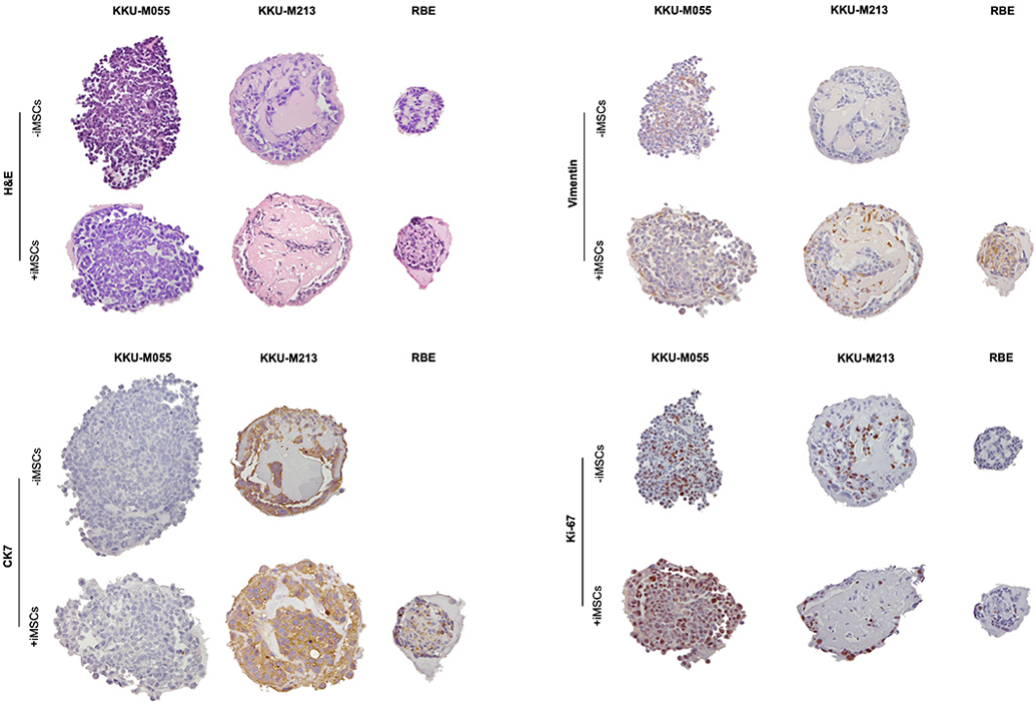
**Pre-clinical 3D models of cholangiocarcinoma, showcasing morphology with H&E staining and protein expression of key markers.** H&E, Haematoxylin and Eosin; iMSC, immortalised mesenchymal stem cell.

**Why did you choose DMM for your paper?**

I chose DMM for my paper because it is a well-regarded journal that publishes research on disease mechanisms using model systems, which aligns well with the focus of my study. My research explores the role of the tumour microenvironment and mesenchymal stem cells in CCA, and DMM provides a relevant platform to share these findings. The journal's emphasis on understanding disease biology through model organisms makes it a suitable venue for my work.

Additionally, DMM is associated with the university where I conducted my PhD research, and my supervisor recommended it as an appropriate journal for my paper. The journal's wide readership, particularly among researchers in cancer biology and disease modelling, ensures that my work will be seen by those who will benefit from it.

Finally, DMM's open-access policy ensures that my research will be accessible to a broad audience. This helps ensure that my findings have the potential for wider impact.

**Given your current role, what challenges do you face and what changes could improve the professional lives of other scientists in this role?**

In my current role in industry, one of the main challenges I face is navigating the transition from a research-focused environment to one where the focus is on clinical trials and translating research into tangible outcomes for patients. This shift requires balancing scientific discovery with the practicalities of regulatory frameworks, timelines, and the need for robust, reproducible data to support clinical decisions. Another challenge is the need to constantly stay up to date with the rapidly evolving landscape of clinical research and industry trends, while also ensuring that the work remains aligned with patient needs.

To improve the professional lives of other scientists in similar roles, there could be a greater emphasis on fostering stronger connections between academic research and industry applications. Encouraging more collaboration and knowledge exchange between these sectors would help bridge the gap between basic science and its clinical implementation.

**What's next for you?**

Next, I plan to continue expanding my role in industry, focusing on the translation of scientific research into clinical applications. I am particularly interested in furthering my involvement in clinical trials, working closely with multidisciplinary teams to bring innovative therapies from the lab to patients. I also aim to deepen my understanding of the regulatory and strategic aspects of drug development.

In the longer term, I hope to take on leadership roles where I can help shape the direction of research and development, fostering collaborations between academia, industry, and clinical settings. Ultimately, my goal is to have a direct, positive impact on patient care through the integration of cutting-edge scientific discoveries into practical, life-changing treatments.

**Tell us something interesting about yourself that wouldn't be on your CV**

Something interesting about me that wouldn't be on my CV is my passion for travelling and exploring different cultures. I find it fascinating to learn about how people from various parts of the world approach challenges and solve problems, and this often inspires me in my scientific work.

Travelling has taught me to embrace diverse perspectives, which I believe is essential in research, where collaboration and thinking beyond conventional boundaries can lead to innovative solutions. It's a way of broadening my understanding of the world, much like science does, by constantly uncovering new insights and connections.

**Who are you grateful to for inspiring and supporting your journey to becoming a scientist?**

I am deeply grateful to my family and partner for their support throughout my journey. A significant part of my inspiration comes from a loved one I lost to cancer. Their resilience and strength during their battle deeply moved me and ignited my determination to contribute to cancer research.

I am also incredibly thankful to my PhD supervisor, who was a true inspiration. Their guidance, passion for science, and dedication to pushing boundaries greatly influenced my growth as a scientist and fuelled my commitment to impactful research.
